# Renal and gastrointestinal complications following tosufloxacin therapy for suspected mycoplasma infection in a 10-year-old child

**DOI:** 10.1007/s13730-025-01007-z

**Published:** 2025-06-17

**Authors:** Shigehiro Sainokami, Hirofumi Watanabe, Shoichiro Kanda, Natsuho Adachi, Hiroyuki Tanaka, Keiichi Takizawa, Naoko Sone, Yuko Kajiho, Akiko Kinumaki, Yoshifumi Morita, Teruhiko Yoshida, Makoto Kurano, Yutaka Harita

**Affiliations:** 1 The Department of Pediatrics, The University of Tokyo Hospital, 7-3-1 Hongo, Bunkyo-Ku, Tokyo 113-8655 Japan; 2 Department of Clinical Laboratory, The University of Tokyo Hospital, 7-3-1 Hongo, Bunkyo-Ku, Tokyo 113-8655 Japan

**Keywords:** Cast nephropathy, Drug-induced tubulointerstitial nephritis, Tosufloxacin

## Abstract

Tosufloxacin, a fluoroquinolone antibiotic, is increasingly prescribed for pediatric patients, particularly for macrolide-resistant *Mycoplasma pneumoniae* infections in Japan. While its efficacy is well-documented, adverse effects such as renal impairment and gastrointestinal symptoms have raised growing concerns. We report a case of a 10-year-old girl who developed sequential symptoms following tosufloxacin administration. On the day after starting tosufloxacin, she experienced right-sided flank pain, followed by the onset of gastrointestinal symptoms, including abdominal pain, diarrhea, and vomiting, on the subsequent day. Contrast-enhanced computed tomography revealed increased fatty tissue opacity surrounding the kidney and duodenal wall thickening, suggestive of localized inflammation extending from the kidney to adjacent structures. Urine microscopy demonstrated needle- and sea urchin-shaped drug crystals, strongly implicating tosufloxacin in the development of renal and gastrointestinal symptoms. This case provides the first evidence suggesting that kidney inflammation may contribute to gastrointestinal symptom onset via localized inflammatory extension. The patient’s symptoms resolved promptly with the discontinuation of tosufloxacin and supportive care. This report underscores the importance of monitoring pediatric patients for renal and gastrointestinal adverse effects following tosufloxacin administration.

## Introduction

Mycoplasma pneumonia is a type of community-acquired pneumonia that predominantly affects school-aged children and young adults. The causative agent, *Mycoplasma pneumoniae (M. pneumoniae)*, is distinguished by its small size and absence of a cell wall. Due to this unique feature, β-lactam antibiotics, including penicillins and cephalosporins, which act by inhibiting cell wall synthesis, are ineffective against *M. pneumoniae*. Consequently, macrolides are regarded as the first-line treatment. However, the increasing prevalence of macrolide-resistant *M. pneumoniae* (MRMP) has emerged as a significant clinical concern in recent years.

Tosufloxacin, a fluoroquinolone antibiotic, is currently approved for use in a few countries, including Japan, South Korea, and other Asian nations. In Japan, its use was expanded to include pediatric patients in 2010 and approved for Mycoplasma infections in 2017. As of 2025, Japan remains the only country, where its prescription is permitted for pediatric patients. With the increasing prevalence of infections caused by MRMP, the use of tosufloxacin has grown in Japan. Although its clinical efficacy has been well-documented, concerns regarding various adverse effects associated with tosufloxacin are increasingly being recognized [1].

We report a case of a pediatric patient who developed renal impairment and gastrointestinal symptoms, which were ultimately attributed to adverse effects of tosufloxacin based on a detailed medical history and findings from urine microscopy. In addition, contrast-enhanced abdominal computed tomography (CT) revealed distinctive features, which are described in this report.

## Case report

A 10-year-old girl presented with right-sided abdominal pain and diarrhea as her chief complaints. She had no significant past medical or family history. Three days prior to hospitalization, she developed fever, sore throat, and cough during the night. As these symptoms persisted, she visited a local clinic 2 days before admission. Although a rapid antigen test for *M. pneumoniae* was negative, the physician diagnosed her with acute bronchitis likely caused by *M. pneumoniae* based on the local outbreak situation and a history of close contact with her brother, who had been experiencing persistent high fever for a week. She was prescribed tosufloxacin at a dose of 450 mg (13 mg/kg).

On the following day, the patient developed right-sided flank pain and returned to the clinic. The pain was initially considered to be due to constipation, and magnesium oxide was prescribed. However, after administration of magnesium oxide, the patient developed diarrhea the next day, leading to the discontinuation of the laxative. Despite discontinuation, the diarrhea persisted and was accompanied by nausea and vomiting.

On the day of admission, as her symptoms persisted, the patient revisited the clinic. Suspecting that her symptoms might be drug-induced, the physician discontinued tosufloxacin. However, her symptoms failed to improve after returning home, leading her to present to the emergency department of our hospital.

On admission, the patient’s body temperature was 37.7 °C, with a heart rate of 100 beats per minute and an SpO₂ of 97% on room air. She was alert and oriented. Abdominal examination revealed a flat and soft abdomen with no evidence of increased or decreased bowel sounds. However, spontaneous pain, tenderness, and rebound tenderness were noted in the right flank and lower abdominal regions. In addition, percussion tenderness was observed over the right costovertebral angle (CVA).

Laboratory results on admission revealed a complete blood count (CBC) and coagulation tests within the reference range. Blood biochemistry showed an elevated C-reactive protein (CRP) level of 1.57 mg/dL and an elevated serum creatinine level of 1.09 mg/dL. The estimated glomerular filtration rate (eGFR), calculated using the Japanese pediatric 5-variable formula [2], was 49 mL/min/1.73 m^2^, indicating impaired renal function. Urinalysis revealed no evidence of pyuria, with an absence of white blood cells in the urine (Table 1). The urine pH was 5.5. The calcium-to-creatinine ratio was 0.01 g/g·Cr, and the N-acetyl-β-D-glucosaminidase (NAG)-to-creatinine ratio was 13.4 IU/g·Cr. Urinary sediment examination showed 50–99 hyaline casts, 10–19 epithelial casts, and 1–4 granular casts per whole field (WF), suggesting tubular involvement.

**Table 1:** Laboratory tests on admission

Parameters	Value	Reference range
Complete blood count		
White-cell count (per μL)	5,300	4,100–1,5000
Haemoglobin (g/dL)	13.7	11.5–14.4
Platelet count (per μL)	273,000	180,000–580,000
Coagulation tests		
PT-INR	1.09	0.90–1.15
APTT (sec)	24.5	26.9–38.1
D-dimer (μg/mL)	0.5	< 1.0
Serum chemistry		
Total protein (g/dL)	6.6	6.2–7.7
Albumin (g/dL)	3.9	3.6–4.7
Sodium (mmol/litre)	140	137–144
Potassium (mmol/litre)	4.2	3.6–4.7
Chloride (mmol/litre)	107	101–110
Urea nitrogen (mg/dL)	23.9	6.6–19.6
Creatinine (mg/dL)	1.09	0.28–0.37
C-reactive protein (mg/dL)	1.57	< 0.3
Lactate dehydrogenase (U/litre)	309	175–320
Alanine aminotransferase (U/litre)	13	9–27
Aspartate aminotransferase (U/litre)	31	24–44
Urinalysis		
pH	5.5	5.0–7.5
Specific gravity	1.005	1.005–1.030
Ketones	Negative	Negative
Nitrite	Negative	Negative
Blood	Negative	Negative
Protein	±	Negative
WBC	Negative	Negative
Ca/Cre (g/g·Cr)	0.01	0.04–0.28
NAG/Cre (IU/g·Cr)	13.4	1.0–6.3
Hyaline casts (/whole field)	50–99	0
Epithelial casts (/whole field)	10–19	0
Granular casts (/whole field)	1–4	0

*APTT* activated partial thromboplastin time, *PT-INR* prothrombin time–international normalized ratio

Given the tenderness and rebound tenderness in the right lower abdomen, the possibility of acute appendicitis was considered, and an abdominal ultrasound was performed. However, no enlargement of the appendix was observed. Instead, Grade 1 hydronephrosis in the right kidney and punctate hyperechoic areas within the renal pelvis were identified. No structural abnormalities of the kidneys were observed, and there were no findings suggestive of urinary tract obstruction. The renal cortical echogenicity was within normal limits, and no focal areas of reduced renal perfusion were clearly identified on ultrasound. During the examination, significant tenderness was elicited when the probe was applied to the affected area.

**Fig. 1:**
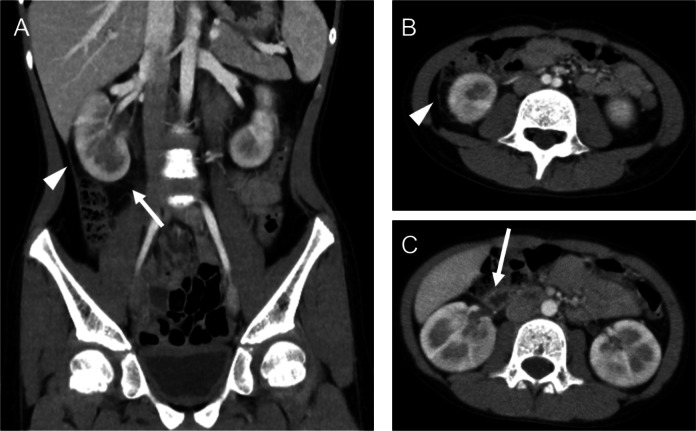
Contrast-enhanced abdominal CT image of the patient. **A** Coronal contrast-enhanced abdominal CT scan of the patient. The arrowhead indicates peritoneal thickening in the right paracolic gutter, while the arrow denotes fat stranding around the right kidney. **B** Axial contrast-enhanced abdominal CT scan of the patient. The arrowhead shows peritoneal thickening in the right paracolic gutter. **C** Axial contrast-enhanced abdominal CT scan of the patient. The duodenal wall appears thickened (arrow)

As pyuria was absent on urinalysis, an abdominal contrast-enhanced CT scan was performed to evaluate for acute focal bacterial nephritis. The CT scan did not demonstrate any hypo enhanced areas within the kidney, which are characteristic of acute focal bacterial nephritis. On the other hand, peritoneal thickening in the right paracolic gutter and fat stranding around the right kidney were observed (Fig. 1A, B). In addition, thickening of the adjacent duodenal wall was observed (Fig. 1C). These findings were suggestive of localized peritonitis, likely secondary to inflammation extending from the right kidney.

In the absence of findings strongly indicative of a bacterial infection, the differential diagnosis included acute enteritis and adverse effects of tosufloxacin. The patient was admitted for observation without the initiation of antibiotics.

By the day after admission, her fever had resolved, and the spontaneous abdominal pain in the right abdomen showed mild improvement. By the third day of hospitalization, the abdominal pain had completely subsided, and her serum creatinine level improved to 0.55 mg/dL, with an eGFR of 86 mL/min/1.73 m^2^.

**Fig. 2:**
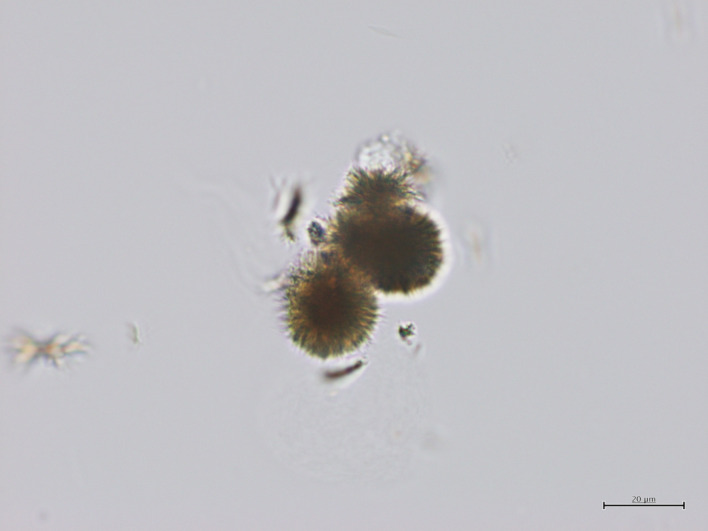
Drug crystals in the patient’s urine observed under microscopy (× 400 magnification) Needle-shaped and sea urchin-shaped crystals were observed in the patient’s urine. These crystals are characteristic of tosufloxacin. The scale bar represents 20 μm

Urine microscopy could not be performed on the day of admission or the following day due to the weekend. However, on the third day of hospitalization, microscopy of the urine sample collected on admission revealed numerous needle-shaped and sea urchin-shaped crystals, findings consistent with drug crystals associated with tosufloxacin (Fig. 2). Under low magnification (× 100), approximately 10–20 crystals per field of view were observed in the sample collected on admission. A small amount of crystals (approximately 1 crystal per several fields of view under low magnification) was observed in the urine sample collected on the second day of hospitalization, while no crystals were detected in the sample collected on the third day. Based on these findings, the abdominal symptoms and renal impairment were diagnosed as adverse effects of tosufloxacin.

By the fourth day of hospitalization, the CVA tenderness had resolved, and the patient was discharged. At follow-up visits after discharge, she remained asymptomatic, with no signs of renal impairment or abdominal symptoms.

## Discussion

At the initial presentation to our hospital, the differential diagnoses based on the patient’s abdominal symptoms and physical findings included acute appendicitis, acute pyelonephritis, acute focal bacterial nephritis, and acute enteritis. After comprehensive evaluations, including several imaging studies, the identification of drug crystals in the urine, along with the spontaneous resolution of abdominal symptoms and renal impairment, led to the diagnosis of adverse effects caused by tosufloxacin.

The use of tosufloxacin has been increasing in recent years, accompanied by a rise in reports of interstitial nephritis associated with this drug, making it one of the leading causative agents of interstitial nephritis [3]. Okada et al. were the first to suggest that tosufloxacin-induced renal impairment is generally caused by interstitial nephritis due to drug crystal formation [4], and this mechanism is also likely in the present case. Recently, Ito et al. performed a renal biopsy on a patient with renal dysfunction induced by tosufloxacin. Using electron microscopy and mass spectrometry analysis, they revealed the presence of numerous crystals with sharp and oval structures in the renal interstitial areas [5].

Notably, an increasing number of cases have been reported in pediatric patients as well, experiencing adverse effects such as renal impairment linked to tosufloxacin use [6–9]. Among pediatric patients without underlying medical conditions, cases are more commonly reported in school-aged children and older. Such cases have also been observed in infants, though less frequently. Most diagnoses are made within 1–3 days after the administration of tosufloxacin. One contributing factor is the increasing number of tosufloxacin prescriptions for children. In Japan, tosufloxacin became the first fluoroquinolone approved for pediatric use in 2010, and its indications were expanded to include *M. pneumoniae* infections in 2017. Another possible reason for these reports is that children may be more prone than adults to developing needle-shaped drug crystals. A Study indicates that the detection rate of needle-shaped crystals relative to the number of prescriptions is eight times higher in children than in adults [1]. Children are also more susceptible to dehydration due to infection-related fever and vomiting, which can lead to urine concentration and promote crystal formation. This case underscores the need for careful monitoring of pediatric patients prescribed tosufloxacin, especially for potential adverse effects, such as crystalluria and renal impairment. Awareness of these risks is critical to ensuring prompt diagnosis and intervention.

In addition to renal impairment, the patient exhibited gastrointestinal symptoms, such as abdominal pain and vomiting. Previous reports have also shown that many cases of adverse effects associated with tosufloxacin involve a combination of renal impairment and gastrointestinal symptoms [6–8], similar to what was observed here. Tosufloxacin, as an antibiotic, can disrupt the balance of the intestinal microbiota, which may lead to gastrointestinal symptoms. However, given the frequent concurrence of renal impairment and gastrointestinal symptoms, some reports have explored their relationship. For instance, dehydration caused by vomiting and diarrhea may increase the concentration of the drug in the blood and urine, promoting crystal formation [7]. This suggests that gastrointestinal symptoms might exacerbate renal impairment. On the other hand, in this case, imaging with contrast-enhanced abdominal CT revealed increased density of the perirenal fat tissue, localized peritonitis surrounding the kidney, and thickening of the duodenal wall on the same side as the patient’s flank pain. These findings suggest that inflammation in the kidney may have extended to nearby structures, particularly the gastrointestinal tract. This hypothesis aligns with the clinical course, as vomiting began the day after the onset of lower back pain.

In this case, the severity of gastrointestinal symptoms and renal impairment was greatest when the quantity of urinary crystals was high and improved as the crystal count decreased. These findings suggest that gastrointestinal symptoms and renal impairment might be associated with the precipitation of urinary crystals. Similar cases have been reported in which the presence of urinary crystals was accompanied by renal impairment and gastrointestinal symptoms [6–8]. However, there are also reports of cases, where urinary crystals were observed without any associated symptoms or abnormalities in blood or urine tests [1]. This suggests that when the quantity of urinary crystals is minimal, clinical symptoms or laboratory abnormalities may not manifest.

In summary, tosufloxacin is a relatively new fluoroquinolone antibiotic that has been increasingly used in recent years in several Asian countries, including Japan. While it demonstrates high efficacy, there is a growing number of case reports of pediatric patients experiencing adverse effects, such as renal impairment and abdominal symptoms, as seen in this case. It is important to accumulate data on cases presenting with adverse effects to analyze their etiology and pathophysiology. In addition, in clinical practice, careful attention should be paid to preventing dehydration when prescribing tosufloxacin. Furthermore, patients and their caregivers should be advised to ensure adequate hydration and to promptly report any signs of abdominal or flank pain when antibiotics like tosufloxacin are prescribed.
